# Hydrogen Sulfide Increases Nitric Oxide Production and Subsequent S-Nitrosylation in Endothelial Cells

**DOI:** 10.1155/2014/480387

**Published:** 2014-05-21

**Authors:** Ping-Ho Chen, Yaw-Syan Fu, Yun-Ming Wang, Kun-Han Yang, Danny Ling Wang, Bin Huang

**Affiliations:** ^1^School of Dentistry, College of Dental Medicine, Kaohsiung Medical University, Kaohsiung 80708, Taiwan; ^2^Department of Biomedical Science and Environmental Biology, College of Life Science, Kaohsiung Medical University, No. 100, Shihchuan 1st Road, San Ming District, Kaohsiung 80708, Taiwan; ^3^Department of Biological Science and Technology, Institute of Molecular Medicine and Bioengineering, National Chiao Tung University, Hsinchu 30068, Taiwan; ^4^Institute of Biotechnology, National Cheng Kung University, Tainan 70101, Taiwan; ^5^Institute of Medical Science, College of Medicine, Tzu Chi University, Hualien County 97004, Taiwan; ^6^Department of Biological Sciences, National Sun Yat-Sen University, Kaohsiung 80424, Taiwan

## Abstract

Hydrogen sulfide (H_2_S) and nitric oxide (NO), two endogenous gaseous molecules in endothelial cells, got increased attention with respect to their protective roles in the cardiovascular system. However, the details of the signaling pathways between H_2_S and NO in endothelia cells remain unclear. In this study, a treatment with NaHS profoundly increased the expression and the activity of endothelial nitric oxide synthase. Elevated gaseous NO levels were observed by a novel and specific fluorescent probe, 5-amino-2-(6-hydroxy-3-oxo-3H-xanthen-9-yl)benzoic acid methyl ester (FA-OMe), and quantified by flow cytometry. Further study indicated an increase of upstream regulator for eNOS activation, AMP-activated protein kinase (AMPK), and protein kinase B (Akt). By using a biotin switch, the level of NO-mediated protein S-nitrosylation was also enhanced. However, with the addition of the NO donor, NOC-18, the expressions of cystathionine-**γ**-lyase, cystathionine-**β**-synthase, and 3-mercaptopyruvate sulfurtransferase were not changed. The level of H_2_S was also monitored by a new designed fluorescent probe, 4-nitro-7-thiocyanatobenz-2-oxa-1,3-diazole (NBD-SCN) with high specificity. Therefore, NO did not reciprocally increase the expression of H_2_S-generating enzymes and the H_2_S level. The present study provides an integrated insight of cellular responses to H_2_S and NO from protein expression to gaseous molecule generation, which indicates the upstream role of H_2_S in modulating NO production and protein S-nitrosylation.

## 1. Introduction

Gas molecules that are produced by cells have been discussed for several decades regarding their protective role in the vascular system. Recently, the diverse physiologic actions of carbon monoxide (CO), nitric oxide (NO), and hydrogen sulfide (H_2_S) and their role in preventing diseases through the mediation of gas-regulating and -sensing mechanisms have attracted a great deal of interest [1]. For example, NO plays an important role in the regulation of the cardiovascular function through a posttranslational protein S-nitrosylation on the cysteine residue [2]. In our previous study, a mechanical shear flow is regarded as protective for endothelial cells (ECs), leading to a series S-nitrosylation of proteins [3]. Investigating the reported mechanisms of NO on EC protection, the NO-mediated S-nitrosylated proteins, such as F1F0-ATPase, reduced the generation of Ca^2+^ and ROS in mitochondria during ischemia/reperfusion injury [4]. NO was also reported to be essential in the prevention of irreversible oxidative stress and finally provided protection from several diseases including cancer, diabetes, and neuron degeneration [5–7].

The toxic effects of hydrogen sulfide (H_2_S) on living organisms have been recognized for nearly 300 years. In recent years, however, interest has been directed towards H_2_S as the third gaseous mediator, which has been shown to exhibit potent vasodilatory activity both* in vitro* and* in vivo*. This is assumed to be realized by opening vascular smooth muscle K_ATP_ channels [8]. Of the three enzymes, cystathionine-**γ**-lyase (CSE), cystathionine-**β**-synthase (CBS), and 3-mercaptopyruvate sulfurtransferase (3-MST) can utilize L-cysteine as a substrate to produce H_2_S. Deficiency of H_2_S-producing enzymes results in some disorders such as homocystinuria, which is characterized by mental retardation, skeletal abnormalities, increased urine homocysteine, increased risks of thromboembolism, and early onset of atherosclerosis [9–11]. H_2_S was also reported to protect against vascular remodeling from endothelial damage [12]. Recently, a signaling molecule for H_2_S was shown to regulate vascular relaxation and angiogenesis via potassium channel S-sulfhydration [13–15].

With a similar physiological function, it is interesting to discuss the interactions between H_2_S and NO in responding stimuli. In the reports cited above, H_2_S and NO in synergy might regulate smooth muscle relaxation and also mitochondrial integration [16, 17]. H_2_S triggers late-phase preconditioning in the postischemic small intestine by an NO- and p38 MAPK-dependent pathway [18]. Despite H_2_S inhibiting NO production in lipopolysaccharide-stimulated macrophages, the H_2_S can also stimulate NO production from other cells [19, 20].

Because of the technical difficulty in detecting gaseous molecules, in the current study, not only monitoring the regulations of theses enzymes but also quantifying the molecules of H_2_S and NO specifically with the new designed fluorescent probes. Therefore, we question here if H_2_S has any upstream role in the regulation of endothelial NO production.

## 2. Materials and Methods

### 2.1. Cell Culture and Drug Treatments

The EAhy 926 cell line was kindly donated by Cora-Jean S. Edgell, University of North Carolina, Chapel Hill. EAhy 926 cells were cultured in DMEM supplemented with fetal bovine serum (FBS, 10%), streptomycin (100 *μ*g/mL), and penicillin (100 U/mL). ECs were replaced by the same medium containing 2% FBS and incubated overnight prior to the experimental NaHS and NOC-18 treatments.

### 2.2. Cell Lysis and Protein Extraction

ECs were washed with cord buffer after treatment [NaCl (0.14 M), KCl (4 mM), glucose (11 mM), and HEPES (10 mM, pH 7.4)] and then lysed with 100 *μ*L of lysis buffer [HEPES (250 mM, pH 7.7), EDTA (1 mM), neocuproine (0.1 mM), and CHAPS (0.4%, w/v)]. After centrifugation, protein supernatant was collected and protein concentrations were determined with BCA assay reagent (Thermo Fisher Scientific Inc., Rockford, IL, USA).

### 2.3. Western Blot Analysis

Forty micrograms of cell lysates with various treatments were mixed with an equal volume of sample buffer [Tris-HCl (62.5 mM, pH 6.8), SDS (3%, w/v), 2-mercaptoethanol (5%, v/v), and glycerol (10%, v/v)] and then separated by SDS-PAGE. The gel was transferred to PVDF membranes (Millipore, MA, USA) and immunoblotted with antibodies: eNOS (1 : 3000; Cell Signaling Tech., MA, USA), peNOS^S1177^ (1 : 2000; Cell Signaling Tech.), AMPK (1 : 3000; Cell Signaling Tech.), Akt (1 : 2000; Cell Signaling Tech.), cystathionine-*γ*-lyase (CSE, 1 : 1000; Abnova, Taipei, Taiwan), cystathionine-**β**-synthase (CBS, 1 : 1000; Abnova), and 3-mercaptopyruvate sulfurtransferase (3-MST, 1 : 1000; Abcam, Cambridge, UK). The membranes were visualized with the SuperSignal West Femto reagent (Thermo Fisher Scientific, IL, USA) on X-ray films. The images from X-ray films were scanned using a digital scanner (Microtek International Inc.) and the density was calculated by the Progenesis Samespots v2.0 software (NonLinear Dynamics, Newcastle, UK).

### 2.4. Application of Fluorescent Probes and Imaging Conditions

For NO detection, 5-amino-2-(6-hydroxy-3-oxo-3H-xanthen-9-yl)benzoic acid methyl ester (FA-OMe) was designed [21]. ECs with NaHS treatment were coincubated with 10 *μ*M of FA-OMe for 4 h prior to imaging. The ECs were washed three times with PBS buffer and then bathed in 2 mL of PBS. The images were obtained by the fluorescence microscope (*λ*ex 460 nm, *λ*em 524 nm; Axiovert 40 CFL, Zeiss). As for 4-nitro-7-thiocyanatobenz-2-oxa-1,3-diazole (NBD-SCN) that was used for detecting H_2_S, the cells were incubated with 5 *μ*M NBD-SCN for 30 min and then subjected to fluorescence microscope (*λ*ex 460 nm, *λ*em 550 nm) [22]. For confocal fluorescence images study, ECs were seeded at a density of 2 × 10^5^ cells/well on cover glasses (24 × 24 mm^2^) and grown for 24 h. The cells with 10 *μ*M of FA-OMe incubation were fixed with 4% formaldehyde solution for 20 min at room temperature. Cell nuclei were stained with 40,6-diamidino-2-phenylindole (DAPI). Cover glasses containing fixed ECs were mounted in a mixture of PBS and glycerol (1 : 1) on a microscopic slide. The cells were observed using a laser scanning confocal imaging system (Olympus FluoView 300) consisting of Olympus BX51 microscope and a 20 mW output argon ion laser.

### 2.5. Flow Cytometry Assay

After fluorescence microscope observation, the ECs were washed twice with PBS and detached by tryptic reaction. ECs were collected by centrifugation and then resuspended in PBS. The fluorescence was immediately measured by the Accuri C6 flow cytometer (BD, NJ, USA) with excitation and emission settings of 488 and 530 nm, respectively. The fluorescence strength was obtained from 1 × 10^4^ cells and statistically calculated from three repeats.

### 2.6. Evaluation of Protein S-Nitrosylation

The cell lysates (200 *μ*g) after NaHS treatment were blocked by methyl methanethiosulfonate (MMTS), reduced by ascorbate, and labelled by biotin according to reported guideline [23]. The biotinylated lysates were then subjected to a reductant-free SDS-PAGE and western blotted with streptavidin-HRP (1 : 3000) following a previous study [24].

## 3. Results and Discussion

### 3.1. NaHS Increased the Protein Level of eNOS and the Phosphorylation on Serine 1177 Residue

**Figure 1:**
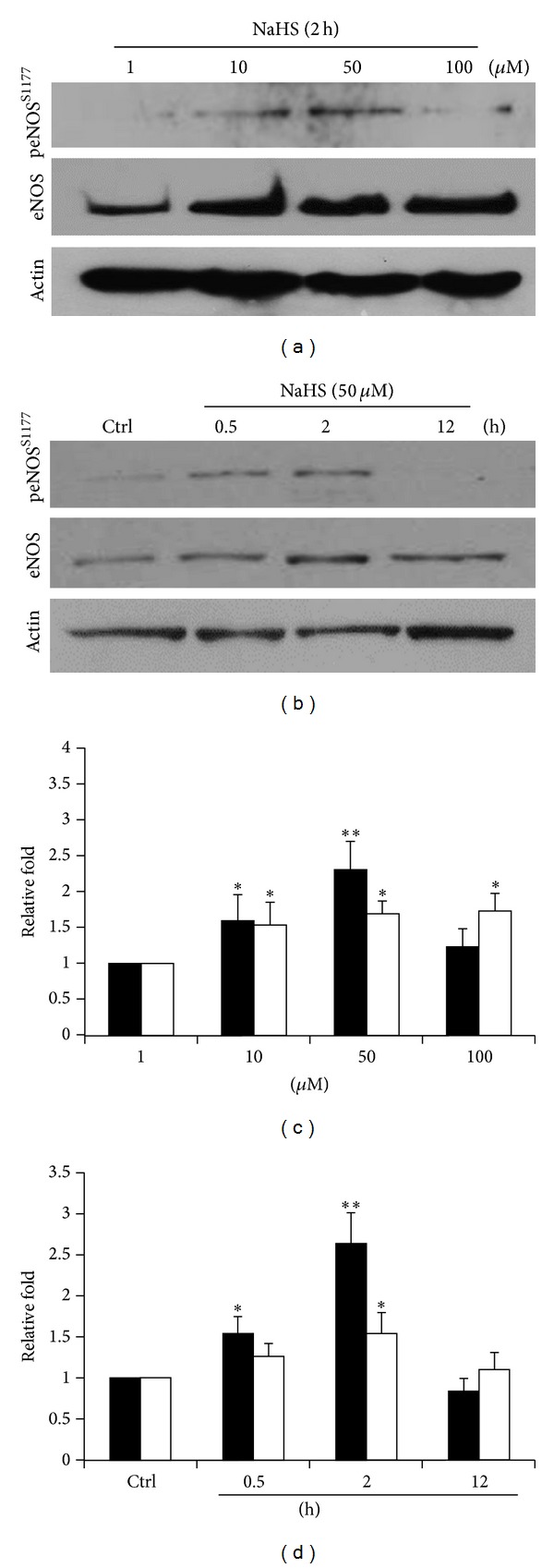
NaHS increases the expression and serine 1177 phosphorylation of eNOS. (a) ECs pretreated by diluting concentrations of NaHS (1 *μ*M, 10 *μ*M, 50 *μ*M, and 100 *μ*M) for 2 h. (b) ECs treated with NaHS (50 *μ*M) for 0.5, 2, and 12 h. Blotted membranes were separately hybridized with eNOS and peNOSS1177 antibodies. ((c) and (d)) Relative folds of protein levels shown as means ± S.E. compared to control. Statistical significance (*P < 0.05; **P < 0.01) analyzed using Fisher's LSD.

Endothelial nitric oxide synthase (eNOS) is responsible for endothelial nitric oxide (NO) production and the enzyme activity is reported to be highly affected by posttranslational phosphorylation on serine 1177 residue (S1177) [25]. In this study, with the treatment of different concentrations of NaHS (1~100 *μ*M), we found that 50 *μ*M of NaHS can significantly enhance both the eNOS expression and the phosphorylation of the serine 1177 residue (peNOS^S1177^) (Figures 1(a) and 1(c)). This concentration conforms well with several vascular research articles [26]. At this concentration, the highest expression level of eNOS and peNOS^S1177^ was observed at 2 hours (Figures 1(b) and 1(d)).

### 3.2. Cellular NO Was Precisely Determined by Specific Fluorescent Probes

**Figure 2:**
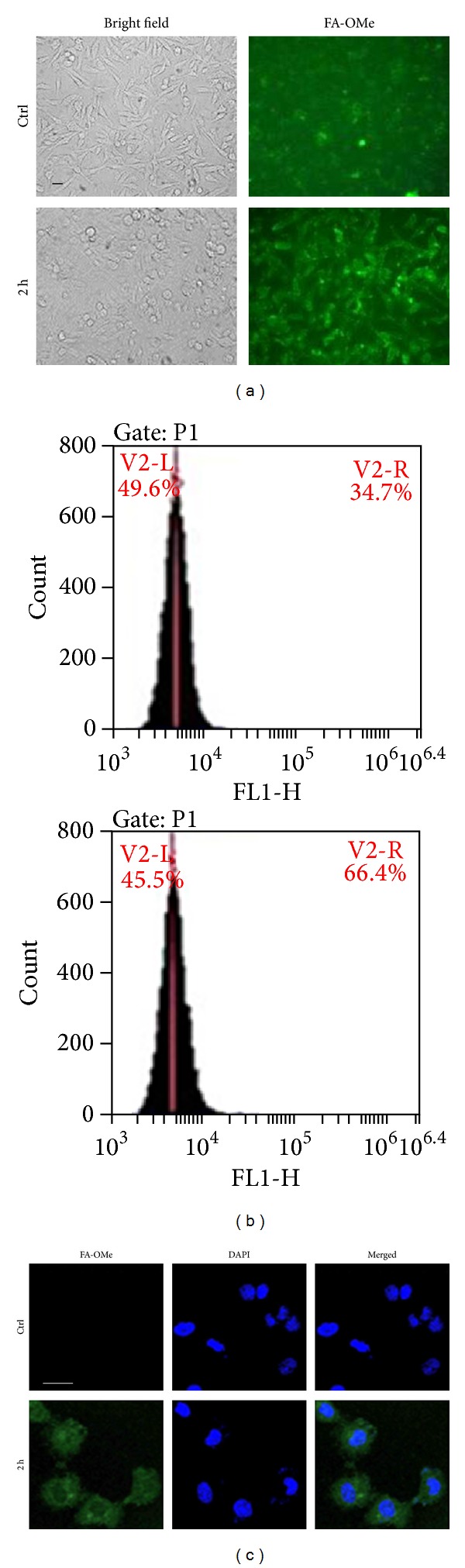
Elevated NO levels monitored by specific fluorescent probes. (a) ECs treated with NaHS (50 *μ*M) for 2 h stained by FA-OMe and observed by fluorescent microscopy. (b) FA-OMe signals calculated by flow cytometry. (c) FA-OMe signals observed by confocal microscopy. Bar = 20 *μ*m.

In addition to the expression of eNOS, the levels of NO molecules were further measured by the specific fluorescent probe FA-OMe. This can distinguish NO and other reactive oxygen species (ROS) from reactive nitrogen species (RNS) [21]. After NaHS treatment, the NO level was increased from 34.7 ± 2.9% to 66.4 ± 3.8% at 2 hours (Figures 2(a) and 2(b)). By using confocal microscopy, despite the fact that the basal fluorescence in the control treatment was difficult to see, the broadly distributed NO was observed in the cytosol and also in the nuclei (Figure 2(c)).

### 3.3. The Expression Profiles of AMPK and Akt in the Presence of H_2_S

**Figure 3:**
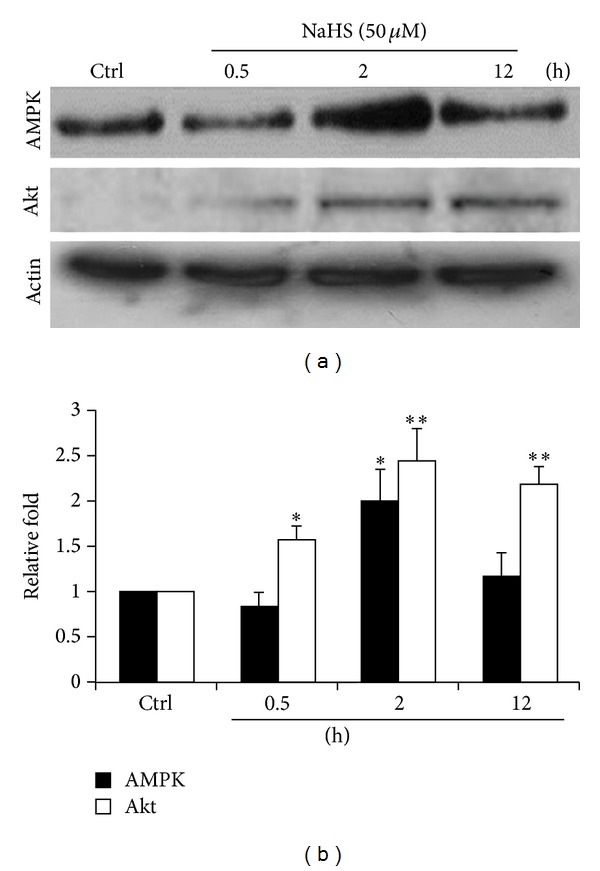
The expression levels of AMPK and Akt in the presence of NaHS. (a) ECs treated with NaHS (50 *μ*M) for 0.5, 2, and 12 h. The blotted membranes hybridized with AMPK antibody. (b) Relative folds of protein levels shown as means ± S.E. compared to control. Statistical significance (*P < 0.05; **P < 0.01) analyzed using Fisher's LSD.

5′ AMP-activated protein kinase (AMPK) is an enzyme that plays a role in cellular energy homeostasis. Besides protein kinase B (Akt), AMPK is also reported to activate eNOS by phosphorylating Ser1177 in response to various stimuli [27]. In the current study, H_2_S increased the protein level of AMPK at 2 h and returned to a basal level at 12 h. However, sustainable expressions of Akt were observed from 0.5 to 12 h (Figures 3(a) and 3(b)). This indicated that H_2_S can stimulate eNOS activity through AMPK and Akt pathways. Similar findings were also reported recently [28, 29].

### 3.4. H_2_S-Increased Bioavailability of NO That Can Enhance Protein S-Nitrosylation

**Figure 4:**
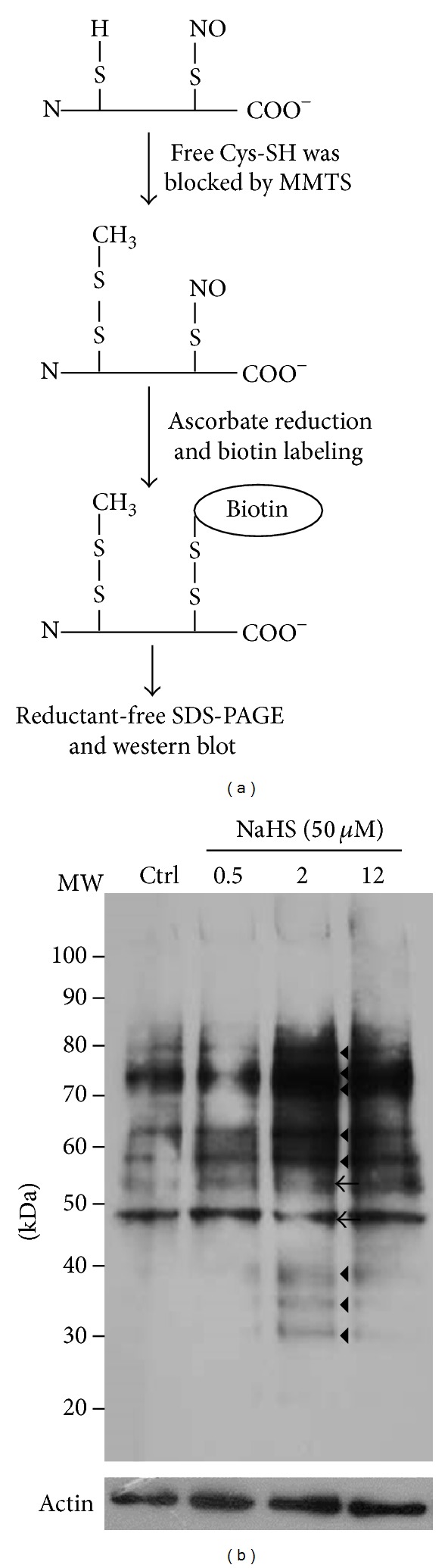
Detection of protein S-nitrosylation. (a) Scheme representing the procedures of modified biotin switch. Biotin-labeled lysates were subjected to SDS-PAGE without any reducing agents in the buffers. (b) ECs lysate (100 *μ*g) treated with NaHS (50 *μ*M) for 0.5, 2, and 12 h separated by SDS-PAGE and the blotted membranes were hybridized with streptavidin-HRP. Triangle indicates proteins with increased S-nitrosylation. Arrow head indicates proteins with decreased S-nitrosylation.

According to previous data, we confirmed that NO level got elevated by H_2_S. Since protein S-nitrosylation/denitrosylation is regarded as important in cardioprotection, its investigation of protein S-nitrosylation is, hence, important for applied medical purposes [2, 3, 30]. By using a modified biotin switch, we could identify at least 8 groups of increased S-nitrosoproteins and 2 groups of decreased S-nitrosoproteins (Figure 4). With the excellent performance in analyzing S-nitrosoproteins, mass spectrometry will be introduced in further identification of these proteins [24].

### 3.5. NO Did Not Reciprocally Increase the Expression of H_2_S-Generating Enzymes and the H_2_S Level

**Figure 5:**
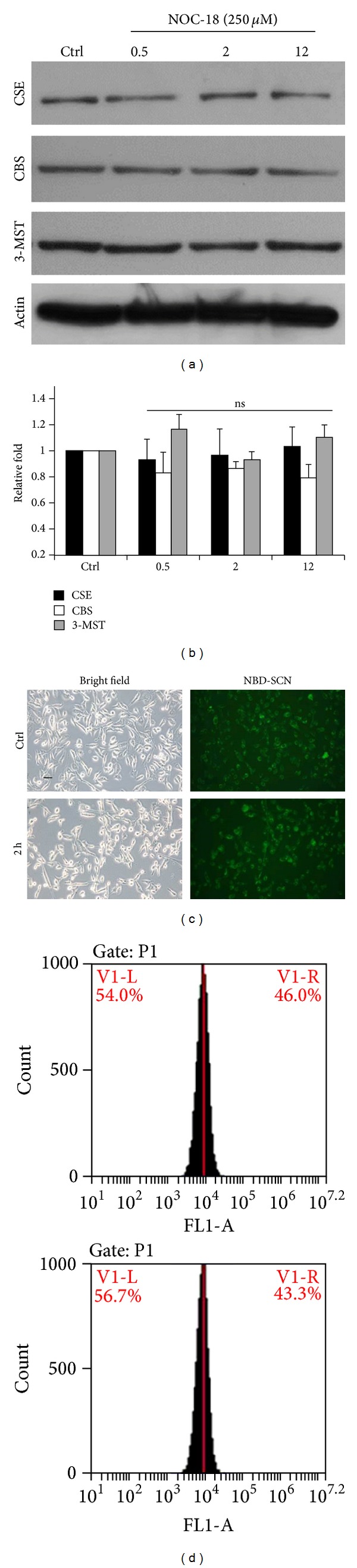
Investigation of H_2_S biosynthesis under the treatment of NO. (a) ECs which treated NO donor, NOC-18 (250 *μ*M), for 0.5, 2, and 12 h were subjected to western blot analysis with CSE, CBS, and 3-MST antibodies. (b) The statistic data showed that no significance (*P* = ns) was observed between treatments. (c) NBD-SCN was applied to detect cellular H_2_S level specifically. (d) Fluorescent signals were calculated by flow cytometry.

After confirming that H_2_S can increase NO at the cellular level, we also examined whether NO can be synchronized at elevated H_2_S levels. As shown in Figure 5, three key enzymes are involved in the cellular H_2_S synthesis: CSE, CBS, and 3-MST. These were not changed by NOC-18 treatment (Figures 5(a) and 5(b)). Although several studies indicated that H_2_S-generating enzymes can also be exerted by NO [31], we could not find differences in our microscopic and flow cytometric analysis (46.0 ± 3.1% and 43.3 ± 4.8% separately) using NBD-SCN fluorescence probe (Figures 5(c) and 5(d)). The reported study demonstrated that H_2_S promotes NO production in ECs via the activation of a cascade of phosphorylation events, starting from p38 MAPK and Akt to eNOS, and this can be through NO-dependent or NO-independent mechanisms cascade. Thus, H_2_S may be a key regulator for angiogenic signalling pathways, whether they required NO or not [32]. This might indicate that NO works as a downstream gaseous transmitter in the endothelium.

## 4. Conclusion

In the present study, hydrogen sulfide increased nitric oxide production. This was not only concluded by studying related enzymes, but also confirmed directly by detecting the final products where NO levels were observed by a novel and specific fluorescent probe, FA-OMe, and quantified by flow cytometry. The level of H_2_S was also monitored by a new designed fluorescent probe, NBD-SCN, with high specificity. The present study provides an integrated insight of cellular responses to two gaseous molecules from protein expression to gaseous molecule generation, which indicates the upstream role of H_2_S in modulating NO production and protein S-nitrosylation.
